# Does age matter in song bird vocal interactions? Results from interactive playback experiments

**DOI:** 10.1186/1742-9994-8-29

**Published:** 2011-11-09

**Authors:** Sarah Kiefer, Constance Scharff, Silke Kipper

**Affiliations:** 1 Institut für Biologie, Verhaltensbiologie, Freie Universität Berlin, Germany

## Abstract

The song of oscines provides an extensively studied model of age-dependent behaviour changes. Male and female receivers might use song characteristics to obtain information about the age of a signaller, which is often related to its quality. Whereas most of the age-dependent song changes have been studied in solo singing, the role of age in vocal interactions is less well understood. We addressed this issue in a playback study with common nightingales (*Luscinia megarhynchos*). Previous studies showed that male nightingales had smaller repertoires in their first year than older males and males adjusted their repertoire towards the most common songs in the breeding population. We now compared vocal interaction patterns in a playback study in 12 one year old and 12 older nightingales (cross-sectional approach). Five of these males were tested both in their first and second breeding season (longitudinal approach). Song duration and latency to respond did not differ between males of different ages in either approach. In the cross-sectional approach, one year old nightingales matched song types twice as often as did older birds. Similarly, in the longitudinal approach all except one bird reduced the number of song type matches in their second season. Individuals tended to overlap songs at higher rates in their second breeding season than in their first. The higher levels of song type matches in the first year and song overlapping by birds in their second year suggest that these are communicative strategies to establish relationships with competing males and/or choosy females.

## Introduction

In most species, behaviour depends on experience and changes with age. Considering communication, an age-differentiated change in signal characteristics might be used by conspecifics as an indicator of age or experience. Alternatively, age might also be actively signaled, i.e. might be the information to be communicated. Age can be one aspect of individual quality with fitness consequences. A long life span might be an honest signal of male genetic quality [1,2] and/or older individuals might have acquired more experience (review in [3,4]).

Male traits are often sexually selected and shaped by female choice [5] and many examples show that females are choosy indeed [review in [6]]. In many different taxa including insects [7,8], fish [9], and mammals [10], females assess the age of males.

Whether and how age and quality might be related has been thoroughly studied in song birds. For example, older males possess better territories [11], take better care of their offspring [12], carry fewer parasites and/or have better immunity [13,14]. Age and reproduction are positively related in several bird species (review in [3]).

Song characteristics such as repertoire size [15,16], repertoire composition [17-19], syllable type consistency [20,21] and vocal performance [22] can all change with age (review in [23]). Most of these age-dependent characteristics were analysed for solo-singing. Only few studies so far investigated age-dependent differences in singing interactions. One year old ortolan buntings (*Emberiza hortulana*) avoided approaching when loudspeakers broadcasted highly threatening songs, but age had no effect on the response to less threatening songs [24]. Adult black redstarts (*Phoenicurus ochruros*) reacted more quickly to playbacks of adult conspecifics than did second year birds [25]. Older banded wrens (*Thryothorus pleurostictus*) overlapped less and responded to a certain song type more often with exactly the same song type (i.e. song type matching, [26]). Several explanations for an age-dependent use of song in interactions have been proposed. Young birds might signal their age particularly in male-male-interactions, e.g. to elicit less aggression (analog to 'delayed plumage maturation', [27]). Alternatively, the competence to apply singing strategies in interactions might be experience-dependent, or only a 'maturation' of the song per se might allow to successfully use it in interactions (e.g. by sharing songs with neighboring males, [28]). Although these explanations are not mutually exclusive, they suggest that age matters in vocal interactions (review in [23]).

Male common nightingales (*Luscinia megarhynchos*) drastically change their repertoire size and composition between their first and second breeding season. Repertoires of one year old birds consist of on average 140 different song types, whereas older birds have repertoires of about 190 different song types [29,30]. After the second breeding season, individual repertoire size and composition remain rather stable [31]. From the first to the second year birds drop song types that are rare on the breeding ground and maintain the 'popular' song types of the population. This leads to an increased similarity to the song repertoire composition of the population [28].

As a consequence of these changes in repertoire size and composition, singing in vocal interactions might differ between individuals of different ages, too. Playback experiments are the method of choice to investigate the use of vocal signals in communicative interactions between males. Playbacks can be described as acoustic simulations of an intrusion of a rival into the territory of a residential male. Nightingales readily interact with a playback, and differentiated response patterns have been described (e.g. [32-36]). Nightingales alternate songs and pauses of similar length. This discontinuous singing style allows different temporal response patterns in singing interactions: Males may take turns singing their songs or overlap each other's songs (review in [37]). The general function of song overlapping as a signal has been recently discussed [38,39]. For nightingales, several playback studies revealed that birds reacted differently in and after a playback that overlapped most of their songs as compared to a playback with alternating or lower levels of overlapping songs [e.g. [36,40-42]]. In addition to temporal response patterns, nightingales may match song types - a behaviour that has been interpreted to be used to address an interactant and/or to signal an aggressive intent (review in [37]). Logue and Forstmeier [43] suggested that song type matching may also facilitate the direct comparison of song quality much better than when song features of different song types have to be compared by listeners.

The aim of this study was to investigate age-related differences in male singing interactions, simulated by nocturnal playback. Given the pronounced differences in solo-singing between one year old and older nightingales, we expected one year old birds to react differently in interactions too. To test this hypothesis we conducted cross-sectional and longitudinal playback experiments and analysed and compared singing responses. If one year old birds reacted less to a simulated vocal intruder, we would expect this to result in longer response latencies, a smaller number of overlapping songs and a smaller number of song type matches by one year old birds compared to older birds.

## Methods

### Subjects and playback procedure

Nocturnal playbacks were conducted in spring 2005 to 2008 (2005: 6 to 26 May; 2006: 30 April to 3 May; 2007: 1 and 3 May; 2008: 8 and 9 May) on a population of individually ringed nightingales in a municipal park in the city of Berlin (see e.g. [28,31,44] for details). The Senatsverwaltung für Stadtentwicklung und Umweltschutz granted permission to capture birds and ringing was done on behalf of the Vogelwarte Radolfzell (Beringungszentrale an der Max-Planck-Forschungsstelle für Ornithologie). All subjects of the study had established territories (as determined by at least 3 consecutive nights of singing from the same song posts). Territory boundaries are mostly well defined by the structure of the park with paths and open meadow areas. This allowed identification of individuals by the location of their territories. Identification was confirmed by reading ring colours the day after the playback trial. The age of the nightingales was determined by characteristic feather features. Birds in their first breeding season usually have characteristic pale tips on their greater secondary coverts and tertials [45-47].

The study subjects were 12 one year old and 12 older birds (2005: n = 6 and 6, 2006: n = 3 and 3, 2008: n = 3 and 3). Five of the one year old birds returned to the study site in the subsequent year and were again tested with a playback (2006: n = 2; 2007: n = 3).

All recordings (during and after playback experiments) were done using Sennheiser ME 80/K3U or ME66/K6 directional microphones with windbreak, and a Sony TCD 5 tape recorder, Sony WMD 6 walkman or a Marantz PMD 660 solid state recorder. We presented the playback songs with a portable CD-player connected to a Sony SRS loudspeaker or a custom-build portable loudspeaker (custom build as suggested in [48], DKA Daniel Kiefer Audio, Heidelberg, Germany). During the playback we recorded the singing of the focal bird with one microphone and the output of the loudspeaker with another one on the stereo channels of the respective recording device.

All playback experiments were conducted at night between 23:30 and 2:00 hrs. We conducted playbacks at the beginning of the breeding season with males engaged in spontaneous singing (indicating an unpaired status [49]). We presented each of the 12 playback strings twice: once to a one year old and once to an older bird, alternating the presentation order between the age groups. These pairs of age groups were tested in close temporal proximity, i.e. within the same night or maximally within two consecutive nights to rule out seasonal effects of different experimental dates between years.

In cases where a neighbouring nightingale was within hearing distance, the playback was started only when this neighbour was silent. The loudspeaker was positioned at the side of the territory opposite to the territory of the closest neighbour in order to avoid interactions with other males during the experiment. The loudspeaker was positioned approximately 15 m or more away from the focal bird's nocturnal song post (presumably the territory border) and was directed towards the focal bird. The experimenter was positioned at least 15 m away from the speaker and the focal bird. Loudness was adjusted to natural nightingale song output (80 dB at a distance of 1 m to the loudspeaker [50]) measured with a precision sound level meter (CEL 314, time constant 125 ms).

### Playback stimuli and playback design

We used high-quality recordings of undisturbed nocturnal song of adult nightingales of our study population from previous years for playbacks. We used songs that were most probably not known to the focal bird, because we avoided songs that were sung in the vicinity of the focal bird in previous years. A start-sequence of 10 randomly chosen consecutive songs was played as a 'prelude' to address the focal bird. Thereafter, we started the playback string. Each of 12 playback strings was assembled from a recording of a different source bird and consisted of a sequence of 70 different songs. The high stereotypy of song type performance in nightingales allows reliable comparisons within and between recordings and birds (see [28,31] for examples). Song types were selected as follows: 50 song types were the same in all playbacks. These were song types that were very common in our study population (i.e., are performed at high rates by many males of both age groups, determined by a population repertoire comparison, Kiefer and Kipper, unpublished data). The additional 20 song types were chosen randomly from the recording of the source bird. The proportion of whistle songs was held constant at 10% in all strings because those have been shown to be a distinct song category [33]. By using song types in a randomized order we aimed to avoid possible sequence effects. As is typical for nightingale song, we played songs with immediate variety i.e., switched to a new song type after each song performed.

Playbacks were prepared and analysed with Avisoft-SASLab Pro software (4.23e, 4.38, 4.40; R. Specht, Berlin). We determined and cut the required song types by visual inspection of spectrograms, filtered background noise (high-pass 0.8 kHz; filter type Butterworth, order 8), normalized songs separately (75% volume) and finally joined 80 single files into one sound file (order randomized).

We presented playback songs interactively: a playback song was started immediately after the end of the focal bird's song which allowed keeping the temporal interaction pattern constant in an alternating mode, independent of the song duration of focal birds. The start-sequence of 10 song types was not replayed interactively and was not included in the data analysis. Playback songs had a duration of 3.05 s ± 0.57 (mean ± SD) and playback trials had an average duration of 766 s ± 61 (mean ± SD).

For the 5 birds that returned in their second breeding season, we additionally conducted a 'longitudinal playback' using the same playback string as the year before.

### Response measures and data analysis

Recordings of playbacks were analysed with Avisoft-SASLab Pro software. The following song responses were determined: During playback we measured the duration of the response song and latencies for each response song to each playback song. Two response styles exist; 'alternating', i.e. a bird does not start his response song before the preceding playback song has ended and 'overlapping', i.e. a bird starts singing before the playback song has ended (overlap: 0.1 s). For our analysis, we treated overlapping and alternating responses separately and counted the number of both. For overlapping songs we measured the latency from the beginning of a playback song to the beginning of the response song whereas latencies of alternating songs were measured from the end of the playback song to the start of the response song. Chance levels for the number of overlapping songs were computed following a calculation presented by Ficken et al. [51]: For each playback trial we determined the cumulative temporal space occupied by playback song ('pb-space') and the entire remaining temporal space ('r-space') during a playback trial. Thus, the probability to sing a song type which overlaps a playback song is 70* pb-space/(pb-space + r-space). Whether the observed numbers of overlapping songs fell below or above chance levels was calculated with Chi-square tests.

Since effects of playbacks might last well beyond the actual interaction, we measured song and pause duration for 2 minutes immediately after playback ended (for 10 pairs only, 2 pairs could not be analysed due to technical problems with recordings).

We determined the number of song type matches, i.e. when the focus bird sang the very same song type that was just played. The likelihood for song type matches was calculated for individuals separately. A song type match by chance should occur with a frequency of 1/repertoire size. Repertoire sizes were calculated following the criteria described in [52]. We analyzed a sequence of 533 complete songs for each bird, which corresponds to a recording length of approx. 1 hour, yielding repertoire curves reaching saturation. For two individuals we had only a sequence of 310 and 428 recorded songs, respectively. For these two birds we estimated the repertoire size as follows: we determined the repertoire size of the bird after 310 songs, chose 5 other birds that had a similar repertoire size at the 310^th ^song, and then averaged the repertoire sizes those 5 birds had at the 533^rd ^song. For the bird with 428 songs we proceeded correspondingly.

The chance level of a song type match during the entire playback trial (70 song types) for each individual is accordingly 70*1/repertoire size. To test for a difference between the observed and the expected number of song type matches we used Wilcoxon signed rank tests for one year old and for older birds. Although this calculation does not take into account how many song types of the playback strings were found in the individual's repertoires, by treating all birds the same (assuming that all individuals possess all playback song types in their repertoires) we selected a conservative measure and enhanced the chance level. Additionally, we compared the number of overlappings and the number of song type matchings between the group of returning (n = 5) and non returning one year old males (n = 7) with a Mann-Whitney U-test to compare whether prospective returners and non-returners differed in these interactions patterns.

For all statistical analyses we used SPSS 15.0. If not otherwise indicated, data are presented as mean ± SD. All tests were conducted two-tailed and, given the small sample sizes, we used exact tests where possible [53]. Using each playback twice (once for a one year old and once for an older bird) resulted in a matched pair design and we tested differences with paired t-tests.

## Results

### Comparison between one year old and older birds

Young and older nightingales responded to playback songs with songs of, on average, identical duration (one year old birds: 2.9 s ± 1.4 (mean ± SD), older nightingales 2.9 s ± 1.5; paired t-test, n = 12, t = 0.17, df = 11, P = 0.87). These values resemble the song duration in solo singing (e.g. [42]).

Alternating songs of one year old birds began 1.3 s ± 1.6 after the end of the playback song, songs of older birds after 1.1 s ± 2.0. When overlapping, one year old birds did so 1.8 s ± 1.3 after a playback song had started, older birds after 2.2 s ± 1.4. Thus, one year old and older nightingales responded with very similar temporal patterns - groups did not differ in latencies of alternating (paired t-test, n = 12, t = 1.23, df = 11, P = 0.24) or of overlapping songs (paired t-test, n = 12, t = 0.20, df = 11, P = 0.85).

In both age groups, seven out of twelve birds overlapped fewer songs than expected by chance (each n = 7, one year old: all χ^2 ^> 5.49, all P < 0.02, older: all χ^2 ^> 10.18, all P < 0.001). Two yearlings (all χ^2 ^> 6.13, all P < 0.013) and one older bird (χ^2 ^= 4.06, P = 0.044) overlapped more songs than expected by chance. The remaining birds of both age groups did overlap at chance levels (one year old: n = 3, all χ^2 ^< 2.89, all P > 0.09, older: n = 4, all χ^2 ^< 3.68, all P > 0.06).

**Figure 1:**
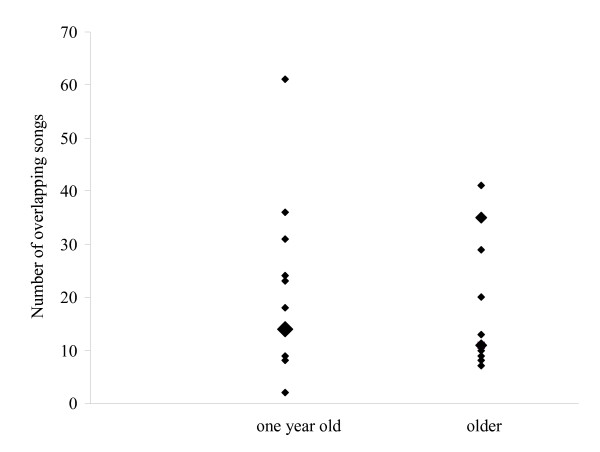
**Number of overlapping songs in the two experimental groups: one year old and older nightingales (each n = 12)**. Increasing symbol size represents 1, 2 and 3 overlapping data points.

The number of overlapped songs did not differ significantly between the two age groups (paired t-test, n = 12, t = 1.08, df = 11, P = 0.30). Older birds overlapped on average 19 ± 12 song types, one year old birds 21 ± 16 (Figure 1).

Song duration and pause duration during the 2 min post-playback phase were also similar between the age groups. Songs lasted 2.9 s ± 1.5 in one year old birds and 3.1 s ± 1.4 in older birds (paired t-test, n = 10, t = -1.55, df = 9, P = 0.16). Pauses lasted 4.7 s ± 3.1 in one year old birds and 4.3 s ± 3.3 in older birds (paired t-test, n = 10, t = 0.34, df = 9, P = 0.74).

**Figure 2:**
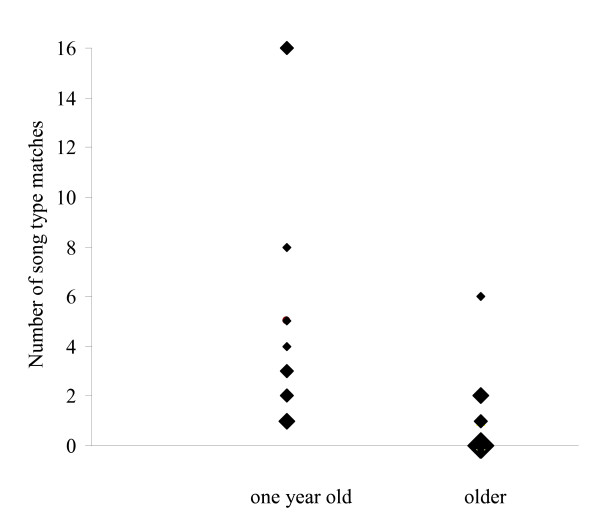
**Number of song type matches in response to playback song types of two experimental groups: one year old and older nightingales (each n = 12)**. Increasing symbol size represents 1, 2, 3 and 6 overlapping data points.

One year old birds had repertoire sizes of 127 ± 29 and older birds 179 ± 24 song types, resembling results from former studies [29]. Chance levels for the number of song type matchings during the playback trial were 0. 58 ± 0.13 for one year old birds and 0.40 ± 0.07 for older birds. Although we found no differences in temporal response patterns, the occurrence of song type matches differed between one year old and older nightingales. Only one year old birds matched song types more often than expected by chance (Wilcoxon signed rank test, each n = 12, one year old birds: W+ = 78, P < 0.05, older birds: W+ = 57, P > 0.05). Younger birds matched song types significantly more often than did older ones (paired t-test, n = 12, t = 2.24, df = 11, P = 0.047). One year old birds matched between 1 and 16 times whereas half of the older birds never matched at all. The remaining matched song types 1 to 6 times (Figure 2).

One year old nightingales matched similar numbers of song types and overlapped songs to an equivalent degree, regardless of whether they returned to the breeding site the next season or not (Mann-Whitney U-Test, each n _returning _= 5, each n _non-returning _= 7, each U > 8, each P > 0.05).

### Longitudinal comparison

The duration of response songs of individual birds was similar in their first (2.8 s ± 0.5) and in their second season (3.0 s ± 0.5). The same held true for latencies of responses to the playback songs. Birds responded after 1.1 s ± 0.4 in their first season and after 1.0 s ± 0.5 in the next season when alternating. When overlapping, one year old birds started their songs on average 2.0 s ± 0.5 after the start of a playback song and after 1.9 s ± 0.6 when they were a year older.

Two one year old nightingales overlapped less than expected by chance (all χ^2 ^> 5.49, all P < 0.02), one overlapped more (χ^2 ^= 6.13, P = 0.01), and two overlapped in the range of chance levels (all χ^2 ^> 0.1, all P > 0.89). In the second year, only one of these birds overlapped less (χ^2 ^= 4.11, P = 0.04), whereas three overlapped more (all χ^2 ^> 9.66, all P < 0.002). One overlapped as often as expected by chance (χ^2 ^= 0.04, P = 0.85). Taken together, individual nightingales tended to overlap more song types when they were in their second (41 ± 17) compared to when they were in their first breeding season (25 ± 9; paired t-test, n = 5, t = 0.1 df = 4, P = 0.06). Singing in the post-playback-phase did not reveal age-dependent differences: The song duration (3.0 s ± 0.5 vs. 3.2 s ± 0.4) and pause duration (4.5 s ± 1.6 vs. 3.5 s ± 1.3) were similar between the first and second season and were in the range of the cross-sectional findings.

**Figure 3:**
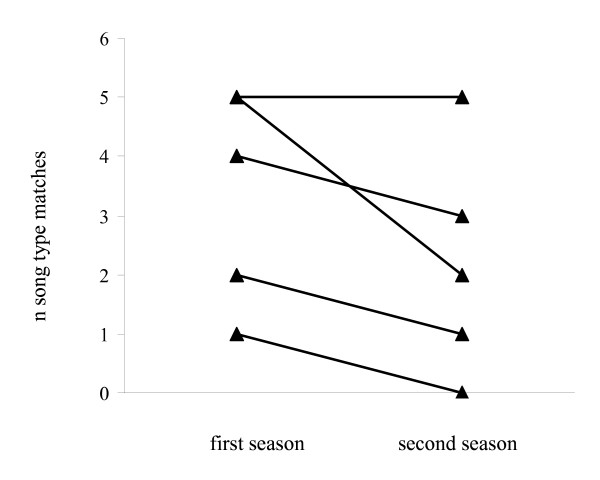
**Number of song type matches in response to playback song types of five birds in their first breeding season and the same birds in their second breeding season**.

All individuals matched the same number or more song types in their first (range 1 to 5) than in their second year (range 0 to 5; Figure 3; paired t-test, n = 5, t = 0.1, df = 4, P = 0.07). Thus, individual differences in matching rates tended to remain rather stable, i.e. birds that song type matched often in their first year did similar in their second and birds that matched less as one year olds matched also less when they were two years old (Spearman's rank correlation: r_s _= 0.82, n = 5, P = 0.08).

## Discussion

One year old nightingales responded differently to a playback than older males: They performed more song type matches. Since song-type matching has been linked to the motivation to escalate a fight [54], we expected older birds to use this response pattern more often, based on the assumption that older birds are of higher quality (suggested e.g. in [44]). This notion is supported by findings that banded wrens that matched the trill type of a playback stimulus indeed approached the speaker closely and matched high and medium performance songs more often than low performance songs [55]. The reason we found more song type matches in younger males may be due to different motivational states: older birds might have been less motivated to match because they can withstand a physical territorial dispute better than one year old birds of lower quality, who may prefer to invest their power into vocal interactions to prevent a physical fight. These considerations are in line with recent findings suggesting that males of lower quality invest more in vocal output. Brumm [56] showed that males of smaller body size and worse condition sang with higher maximum amplitudes and Schmidt et al. [57] demonstrated that males who reacted stronger to playbacks subsequently did not succeed in pairing. Alternatively, one might also consider that older birds are better able to adjust the appropriate level of matching based on prior experiences with neighboring birds, as has been suggested in several neighbor-stranger-studies (e.g. [58,59]).

Another, though not mutually exclusive explanation for fewer song type matches in older birds might be provided by their larger repertoires [29,30] which in turn might make it more challenging to select the adequate song type for matching. Different pairing states of males of the two groups might provide yet another explanation for different matching rates (e.g. [26]). Although we did not systematically determine the pairing status, we consider this explanation least likely because sustained nocturnal singing by all focal birds suggests that they were not paired yet [49], and playback dates did not differ among the age groups.

In contrast to differences in the selection of response song types, the temporal response patterns did not differ between age groups. Both one year old and older nightingales responded with songs of similar duration. Latencies to respond to the playback songs were also similar in both age groups, both for overlapping and for alternating songs. Many birds overlapped significantly above or below chance levels: this suggests that timing of songs might be an informative response pattern (see [38,39]), even though in our playback experiment not all birds reacted in the same direction. This might be taken as a hint that the competence to respond adequately exists already in one year old birds that are comparatively inexperienced in vocal interactions (review in [23]).

Our results on the timing of song responses are in contrast to the behaviour of black redstarts, where older birds reacted faster (starting singing) than did one year old birds [25]. Black redstarts show pronounced delayed plumage maturation and it has been argued that individuals of the species honestly signal their status by both visual and acoustic cues [25]. This is different in our study species because one year old nightingales differ from older ones only by a very subtle variation in feather colouration [45-47] and we do not know whether these visual differences can be detected by the birds. Instead, nightingales show a delayed song maturation since the repertoire size of older birds is profoundly larger than that of one year old birds (this study and [29]). The playback experiment presented here adds an additional age-dependent song difference. Although it did not reveal differences in the timing of vocal interactions between one year old and older birds, we did find a difference on a structural level. Taken together, these findings suggest that nightingales are indeed an example for a species with 'delayed song maturation' that affect several aspects of song (review in [23]).

Alternatively or in addition to maturation, changes in singing with age might reflect the social experience of birds. Nightingales increase and change their repertoire between year one and two towards the most common songs in the population [28]. This has been explained by a social dynamics benefit: higher levels of song sharing can be beneficial for males in terms of e.g. reduced aggression [58] or territory tenure [60] (but see discussion on song type matching above).

Five of the 12 one year old birds returned for a second breeding season, which provided the opportunity to present the same playback they had heard in their first season. This allowed us to track possible changes between first and second breeding season longitudinally on an individual basis. Overall, the findings mirrored the results of the cross-sectional playback experiments: Concerning the duration of response songs and pauses, no changes between the two successive years were detected. The decreased number of song type matches in the second year playback also resembled the findings of the cross-sectional experiments. Interestingly though, the number of overlapping songs tended to be higher in the second than the first year (mean of 41 vs 25). This was different from the result of the cross-sectional playback analysis (mean 21 for first year vs 19 for older birds), in which the 'older' group consisted of 'anything older than one year'. It might be that birds in their second season have a higher propensity to engage in territorial challenges than younger or older birds. The notion that overlapping represents a 'defensive and not offensive threat' in banded wrens should also be considered here. In this species, overlapping occurred at highest rates in one year olds and declined with age [26].

Comparing singing strategies of one year old birds that returned to the breeding site for a second season with birds that did not return might shed light on possible fitness benefits in this respect. However, at least with our limited sample we did not find any differences in singing strategies between these two groups.

In summary, when only considering the duration of songs and the latencies to respond it is impossible to distinguish one year old from older nightingales in a singing interaction. However, one year old birds matched song types more often. The inverse relationship between song type matching and age and the overall relatively low rates of matching raise the possibility that song type matching in a species with large repertoires may have different valence or function than in species with smaller repertoires like the song sparrow [54]. Logue and Forstmeier [43] proposed that song type matching may provide the opportunity to directly compare (similar) signals from different singers. The nightingale seems a promising candidate species to test assumptions derived from this idea by measuring acoustic features of song type matches such as element rates or lengths and consistency of repetitive parts. Treating nocturnally singing nightingales as communication networks with information-seeking listeners such as third-party males or females as important players appears to be a very promising approach in that direction.

## Competing interests

The authors declare that they have no competing interests.

## Authors' contributions

SKie performed the research and analysed data, SKie and SKip wrote the paper, CS critically revised the manuscript. All authors designed the research, provided substantial intellectual contribution, read and approved the final manuscript.
